# New species and records of *Parametriocnemus* Goetghebuer from China (Diptera, Chironomidae)

**DOI:** 10.3897/zookeys.320.4927

**Published:** 2013-07-31

**Authors:** Xing Li, Xiao-long Lin, Xin-hua Wang

**Affiliations:** 1 College of Life Sciences, Nankai University, Tianjin 300071, China

**Keywords:** Chirnomidae, *Parametriocnemus*, new species, new records, China

## Abstract

The Chinese species of *Parametriocnemus* Goetghebuer are reviewed. Two species, *Parametriocnemus fortis* **sp. n.** and *Parametriocnemus vittatus* **sp. n.** are described and illustrated as males, and *Parametriocnemus ornaticornis* (Kieffer), *Parametriocnemus scotti* (Freeman) and *Parametriocnemus brundini* Sinharay & Chaudhuri are recorded from China for the first time. A key to the males of the seven Chinese *Parametriocnemus* speciesis given.

## Introduction

The genus *Parametriocnemus* was described as a subgenus of *Metriocnemus* van der Wulp by Goetgebuer (1933), based on *Metriocnemus stylatus* Kieffer, 1924. It was raised to genus by Brundin (1956). The genus presently include 34 species worldwide. Seven species are recorded from the Oriental Region, 19 from the Palaearctic Region, 6 from the Nearctic Region, 1 from the Neotropical Region, 3 from the Afrotropical Region and 2 from the Australasian Region (Ashe and O’Connor 2012).

Wang (2000) listed two species of *Parametriocnemus* from China, *Parametriocnemus stylatus* and *Parametriocnemus lundbecki*, based on males, while a record of *Parametriocnemus lundbeckii* was treated as dubious. Based on recently collected material from China, two new species are described and three additional species are recorded. A key to the males of the Chinese *Parametriocnemus* is presented.

## Materials and methods

The morphological nomenclature follows Sæther (1980). The material examined was mounted on slides in Canada balsam, following the procedure outlined by Sæther (1969). Measurements are given as ranges.

The types and other material is housed in the College of Life Sciences, Nankai University, China (BDN).

## Species descriptions

### Parametriocnemus brundini

Sinharay & Chaudhuri

http://species-id.net/wiki/Parametriocnemus_brundini

Parametriocnemus brundini Sinharay & Chaudhuri, 1979: 119.

#### Meterial examined.

CHINA: Fujian Province, Daiyun Mountain, 25°41'0.38"N, 118°11'23"E, 1 male, 13.iv.2002, light trap, Z. Liu.

#### Remarks.

The species can be separated from other members of the genus by having a brown body; absence of band on mesonotum; setae on abdominal terga in transverse rows; long anal point; and a triangular inferior volsella. According to Sinharay and Chaudhuri (1979), the color of the India specimen is brown. The Chinese specimen is lighter brown; other differences between specimens from China and India as in Table 1.

**Table 1.** Difference between specimens from China and India of *Parametriocnemus brundini* Sinharay & Chaudhuri, male.

***Parametriocnemus brundini* Sinharay & Chaudhuri**	**Chinese specimens (n=1)**	**India specimens (n=1)**
AR	1.03	1.06
Color of thorax	yellowish	brown
Color of abdomen	yellowish	I–IV brown, rest dark brown
LR_1_	0.82	0.77
LR_3_	0.62	0.44

#### Distribution.

In China the species is known from the Fujian Province in the Oriental region only.

### Parametriocnemus fortis sp. n.

http://zoobank.org/FA0E5DF9-3AD6-430E-BB7A-2BCF02A97084

http://species-id.net/wiki/Parametriocnemus_fortis

Figs 1–3

#### Material examined.

Holotype male (BDN No.007), CHINA: Tibet, Shergmla Mountain, Lulang, 29°56'36"N, 94°47'57"E, 29.ix.1997, light trap, T. Solhøy & J. Skartveit.

#### Diagnostic characters.

The male differs from other members of the genus by having a long, strong anal point, twice as long as gonostylus, and a high HV.

#### Etymology.

From Latin, adjective, *fortis* – meaning strong, referring to the long and strong anal point.

#### Description.

Male (n=1).

Total length 3.03 mm. Wing length 2.08 mm. Total length / wing length 1.46. Wing length / length of profemur 2.59.

*Coloration*. Head and wing light brown. Legs yellow. Thorax and abdomen blackish brown.

*Head*. AR 0.57. Temproal setea 13, including 8 inner verticals, 2 outer verticals and 3 postobitals. Clypeus with 11 setae. Tentorium 185 µm long, 38 µm wide. Palpomeres lost.

**Figures 1–3.**
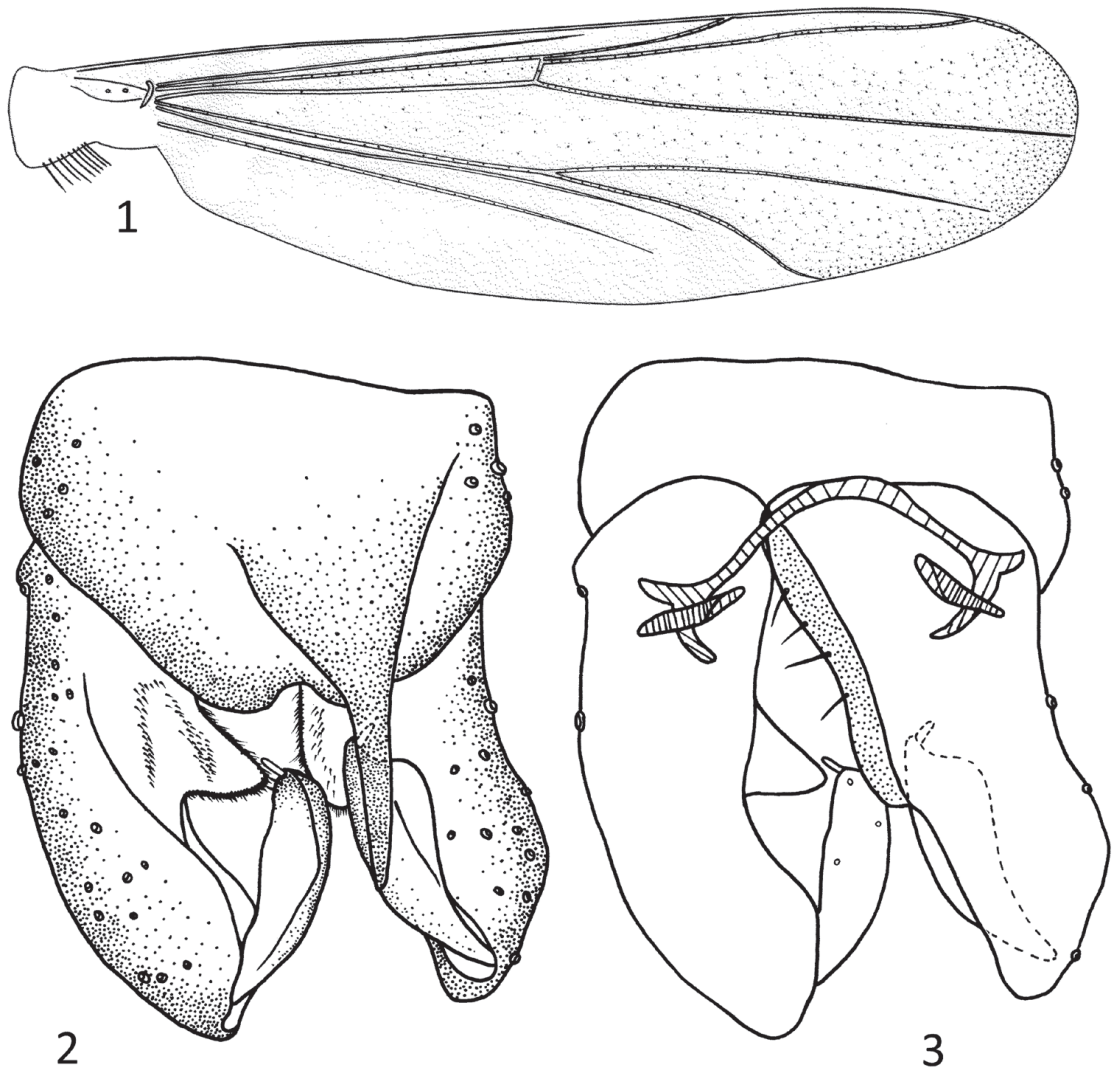
*Parametriocnemus fortis* sp. n., male. **1** wing **2** hypopygium(dorsal view) **3** hypopygium (ventral view).

*Wing* (Fig. 1). Anal lobe reduced. VR 1.06. Costal extension 75 µm long, ending above to very slightly proximal to apex of M_3+4_. Brachiolum with 1 seta, C extension with 5 non-marginal setae, Sc bare, R with 23 setae, R_1_ with 16, R_4+5_ with 37, RM with 1, M with 2, M_1+2_ with 64, M_3+4_ with 39, Cu with 20, Cu_1_ with 28, Pcu with 30, and An with 22 setae. Cell m proximal to RM with 9 setae, r_4+5_ with 162, m_1+2_ with 176, m_3+4_ with 125, an with 2, and cu with 23 setae. Squama with 8 setae.

*Thorax*. Antepronotum with 1 setae. Dorsocentrals 20, acrostichals 3, prealars 6. Scutellum with 7 setae.

*Legs*. Spur of fore tibia 43 µm long, spurs of mid tibia 20 µm and 18 µm long, of hind tibia 43 µm and 25 µm long. Width at apex of mid tibia 40 µm. Comb of 10 setae, shortest seta 30 µm long, longest seta 50 µm long. Lengths (in µm) and proportions of legs as in Table 2.

**Table 2.** Lengths (in µm) and proportions of legs segments of *Parametriocnemus fortis* sp. n., male (n = 1).

	**fe**	**ti**	**ta_1_**	**ta_2_**	**ta_3_**	**ta_4_**
p_1_	800	920	640	–	–	–
p_2_	820	810	420	185	137	93
p_3_	910	990	640	300	–	–
	ta_5_	LR	BV	SV	BR	
p_1_	–	0.70	–	–	–	
p_2_	80	0.52	4.16	–	3.22	
p_3_	–	0.65	–	–	–	

*Hypopygium* (Figs 2–3). Anal point strong, 143 µm long, 88 µm wide at base. Tergite IX including anal point with 5 setae. Laterosternite IX with 4 setae. Pallapodeme 43 µm long, trasverse sternapodeme 60 µm long. Gonocoxite 163 µm long, interifor volsella triangular. Gonostylus 71 µm long, megaseta 14 µm long. HR 2.31, HV 4.30.

#### Remarks.

The species is similar to *Parametriocnemus stylatus* (Kieffer) in the structure of the hypopgium, but can be distinguished by having much stronger anal point.

#### Distribution.

The specimen was collected in Tibet in Palaearctic China.

### Parametriocnemus lundbeckii

(Johannsen)

http://species-id.net/wiki/Parametriocnemus_lundbeckii

Metriocnemus lundbeckii Johannsen, 1905: 302.
Parametriocnemus lundbeckii (Johannsen); Sublette (1967: 5379); Sæther (1969: 115).

#### Material examined.

CHINA: Zhejiang Province, Tianmu Mountain, 30°18'44"N, 119°26'35"E, 7 males, 12.xi.1998, light trap, H. Zhou.

#### Remarks.

The species differs from other members of the genus by having a triangular, broad inferior volsella with bluntly rounded corner. The species is very similar to *Parametriocnemus stylatus* (Kieffer), but differs in the shape of the inferior volsella, and the preapical projection of the gonostylus is much smaller and pointed than that in *Parametriocnemus lundbeckii* (Brundin 1956). According to Sæther (1969), the immature stages seem to be inseparable, and as *Parametriocnemus stylatus* is known to be very variable (see Thienemann 1937) it might be a synonym of *Parametriocnemus lunbdbeckii*.

#### Distribution.

The species has been recorded from the Oriental, Neotropical and Nearctic Regions, and occurs in both of Oriental and Palaearctic China.

### Parametriocnemus ornaticornis

(Kieffer)

http://species-id.net/wiki/Parametriocnemus_ornaticornis

Metriocnumus ornaticornis Kieffer, 1917: 225.
Parametriocnemus ornaticornis (Kieffer); Freeman (1961: 660); Hazra et al. (2002: 45).

#### Meterial examined.

CHINA: Fujian Province, Daiyun Mountain, 25°41'0.38"N, 118°11'23"E, 2 males, 13.ix.2002, light trap, Z. Liu. Yunnan Procince, Eryuan County, Meiyou River, 26°6'40"N, 99°57'3"E, 1 male, 24.v.1996, light trap, C. Zhou. Henan Province, Luanchuan County, Lonyuwan National Forest Park, 33°46'41"N, 111°37'45"E, 1 male, 10.vii.1996, J. Li. Hunan Province, Yanling county, Taoyuan Hole, 26°25'21"N, 113°40'9"E, 1 male, 16.vii.2004, light trap, C. Yan.

#### Remarks.

The species can be separated from other members of the genus by having a comparatively low AR (0.31–0.46), macrotrichiae forming streaks in the apical half of the wing, squama with 4–5 setae, and anal point with bare apex and 3–4 setae on each side. The species was described from Australia by Kieffer (1917) as a member of *Metriocnemus* van der Wulp, and was transferred to *Parametriocnemus* Goetghebuer by Freeman (1961). The specimens from China are in accordance with the original description, but have a lower LR and AR than specimens from India. The differences between specimens from China and India are listed in Table 3.

**Table 3.** Difference between specimens from China and India of *Parametriocnemus ornaticornis* (Kieffer), male.

***Parametriocnemus ornaticornis* (Kieffer)**	**Chinese specimens (n=5)**	**India specimens (n=4)**
AR	0.31–0.46	0.41–0.46
TL	1.44–2.42	2.25–2.52
TL/WL	1.04–1.75	1.77–1.88
LR_1_	0.69–0.83	0.81–0.82
LR_2_	0.49–0.55	0.55–0.56
LR_3_	0.58–0.63	0.60–0.63
SV_1_	2.03–2.74	3.08–3.14
SV_2_	3.93–4.42	5.09–5.13
SV_3_	3.06–3.39	4.00–4.11
BR_1_	0.77–2.13	2.20–2.25
BR_2_	2.00–2.75	2.40–4.00
BR_3_	2.63–3.56	3.00–3.83

#### Distribution.

The species has been recorded from Australia (Kieffer 1917) and India (Hazra et al. 2002). It occurs in both Oriental and Palaearctic China.

### Parametriocnemus scotti

(Freeman)

http://species-id.net/wiki/Parametriocnemus_scotti

Metriocnemus scotti Freeman, 1953: 129.
Parametriocnemus scotti (Freeman); Lehmann (1979: 42).

#### Material examined.

Ningxia Hui Autonomous Region, Liupan Mountain, 35°47'22"N, 106°17'36"E, 1 male, 9.viii.1987, light trap, X. Wang. Zhejiang Province, Taizhou City, Xianju County, Shenxianju Mountain, 28°42'17"N, 120°36'38"E, 1 male, 14.iv.2011, sweep net, X. Lin.

#### Remarks.

The species can be separated from other members of the genus by having a short anal point with bare, rounded apex; triangular inferior volsella, and clavate antenna with numerous curved bristles apically. The species is similar to *Parametriocnemus brundini* Sinharay et Chaudhuri in the structure of the hypopgium; it has a short anal point with bare apex, while that in *Parametriocnemus brundini* is longer. The species was redescribed and figured by Lehmann (1979, figs 124–125). However, no data for the legs was given; lengths (in µm) and proportions of the legs of the Chinese specimens are therefore given in Table 4.

**Table 4.** Lengths (in µm) and proportions of legs segments of Chinese specimens of *Parametriocnemus scotti* (Freeman), male (n=2).

	**fe**	**ti**	**ta_1_**	**ta_2_**	**ta_3_**	ta_4_
p_1_	610–680	690–700	500–560	215–300	194–218	138–141
p_2_	590–700	560–750	282–490	132–245	97–193	62–105
p_3_	660–720	720–740	430–480	194–248	150–178	88–105
	ta_5_	LR	BV	SV	BR	
p_1_	98–128	0.71–0.81	2.47–2.64	2.45–2.62	1.10–2.75	
p_2_	58–93	0.50–0.65	3.06–3.50	2.96–4.08	2.20–3.0	
p_3_	40–78	0.60–0.65	3.19–3.84	3.05–3.21	2.67–6.13	

#### Distrubution.

The species has been recorded from Ethiopia (Abyssinia), Kenya, Uganda and Zimbabwe (Rhodesia) in the Afrotropical Region (Lehmann 1979), and it occurs in both Oriental and Palaearctic China.

### Parametriocnemus stylatus

(Kieffer)

http://species-id.net/wiki/Parametriocnemus_stylatus

Figs 4–6

Metriocnemus stylatus Kieffer, 1924: 97.
Parametriocnemus stylatus (Kieffer); Wang (2000: 638).

#### Meterial examined.

CHINA: Fujian Province, Wuyi Mountain, 27°43'46"N, 118°1'52"E, 1 male, 24.iv.2002, light trap, W. Bu. Fujian Province, Wuyi Mountain, 27°43'46"N, 118°1'52"E, 1 female, 30.viii.1993, light trap, X. Wang. Fujian Province, Shanghang Country, Buyun Mountain, Shiyankeng, 25°15'59"N, 116°51'50"E, 1 male, 6.v.1993, light trap, X. Wang. Beijing City, Huairong District, 40°19'15"N, 116°37'59"E, 2 males, 15.x.1994, light trap, X. Wang. Henan Province, Luanchuan County, Longyuwan National Forest Park, 33°46'41"N, 111°37'45"E, 1 male, 10.vii.1996, light trap, J. Li. Sichuan Province, Yaan City, Yajiang River, Sandaoqiao Town, 29°53'48"N, 103°10'19"E, 1 male, 9.vi.1996, light trap, X. Wang. Sichuang Province, Yanan City, Yajiang County, 29°53'48"N, 103°10'19"E, 1 female, 14.vi.1996, light trap, X. Wang. Shannxi Province, Zhouzhi County, Banfangzi Town, 34°9'47"N, 108°13'19"E, 1 male, 7.viii.1994, light trap. X. Wang. Zhejiang Province, Tianmu Mountain, 30°18'44"N, 119°26'35"E, 2 males, 12.xi.1998, light trap. X. Wang. Yunnan Province, Eryuan County, Niujie Town, Futian Village, Meigong Stream, 26°6'40"N, 99°57'3"E, 5 males, 24.v.1996, light trap, C. Zhou. Guizhou Province, Daozhen County, Dasha River, 26°38'19"N, 108°3'41"E, 3 larvae, 23.v.2004, leg, H. Tang.

#### Remarks.

The male differs from other members of the genus by having AR 0.79–1.09, wing membrane with numerous setae, a rather slender gonostylus without projection, robust anal point, and gonocoxite with a broad, subrectangular inferior volsella.

**Figures 4–6.**
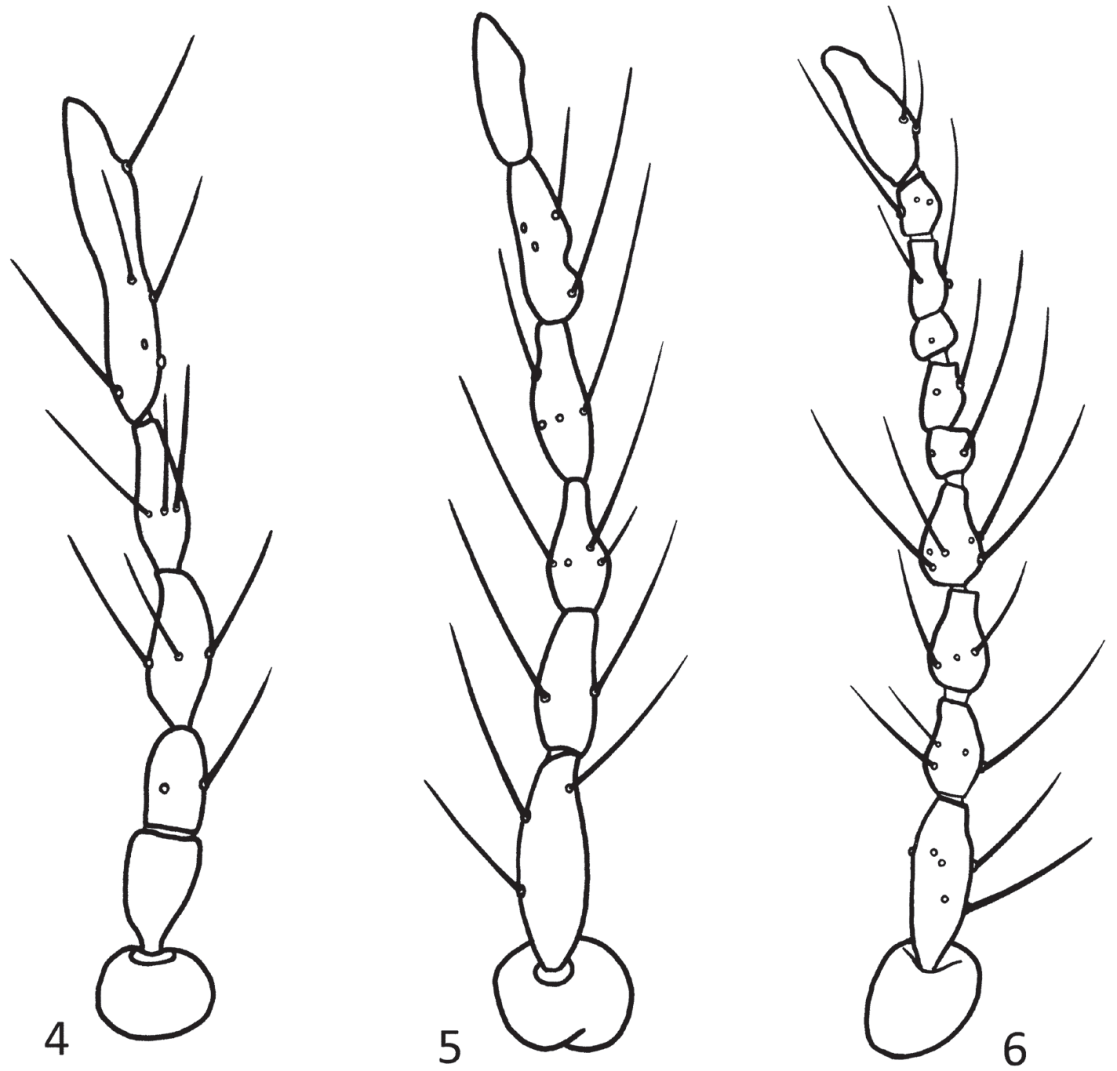
*Parametriocnemus stylatus* (Kieffer), intersex. **4** antenna, five segmented **5** antenna, six segmented; **6** antenna, ten segmented.

According to Wang (2000), five females of this species were collected in the Yunnan Province. After re-examing the specimens, we found that all five specimens apparently are intersexes. One of these intersexes has a 10 segmented antenna, two have a 6 and two a 5 segmented antenna. They are morphologically similar to males, but differ from all species of *Parametricnemus* in structure of the male hypopygium and the female-like antenna, reduced number of setae on the antennal flagellum, and low antennal ratio (Figs 4–6). None of the males from the other localities appear to be intersexes.

#### Distribution.

The species has been recorded from the Palaearctic and Nearctic Regions and occurs in both Oriental and Palaearctic China.

### Parametriocnemus vittatus sp. n.

http://zoobank.org/01BC3723-E3F3-4C4F-8711-DA3897875EBC

http://species-id.net/wiki/Parametriocnemus_vittatus

Figs 7–11

#### Type material.

Holotype male (BDN No.11836), CHINA: Sichuan Province, Shimian County, Sala River, 29°13'40"N, 102°21'34"E, 16.vi.1996, sweep net, X. Wang.

#### Diagnostic characters.

The male differs from other members of the genus by having a low AR, and tergites II–III with brown vita, tergites IV–V with three brown patches, and tergites VI–VII all brown. Ultimate flagellomere is expanded in the middle, tapering toward apex, with 4 long, curved sensilla chaetica subapically.

#### Etymology.

From Latin, noun, *vitta* – meaning ribbon, referring to tergites II–VII having brown vita.

#### Description.

Male (n=1).

Total length 2.55 mm. Wing length 1.43 mm. Total length / wing length 1.58. Wing length / length of profemur 2.30.

**Figures 7–11.**
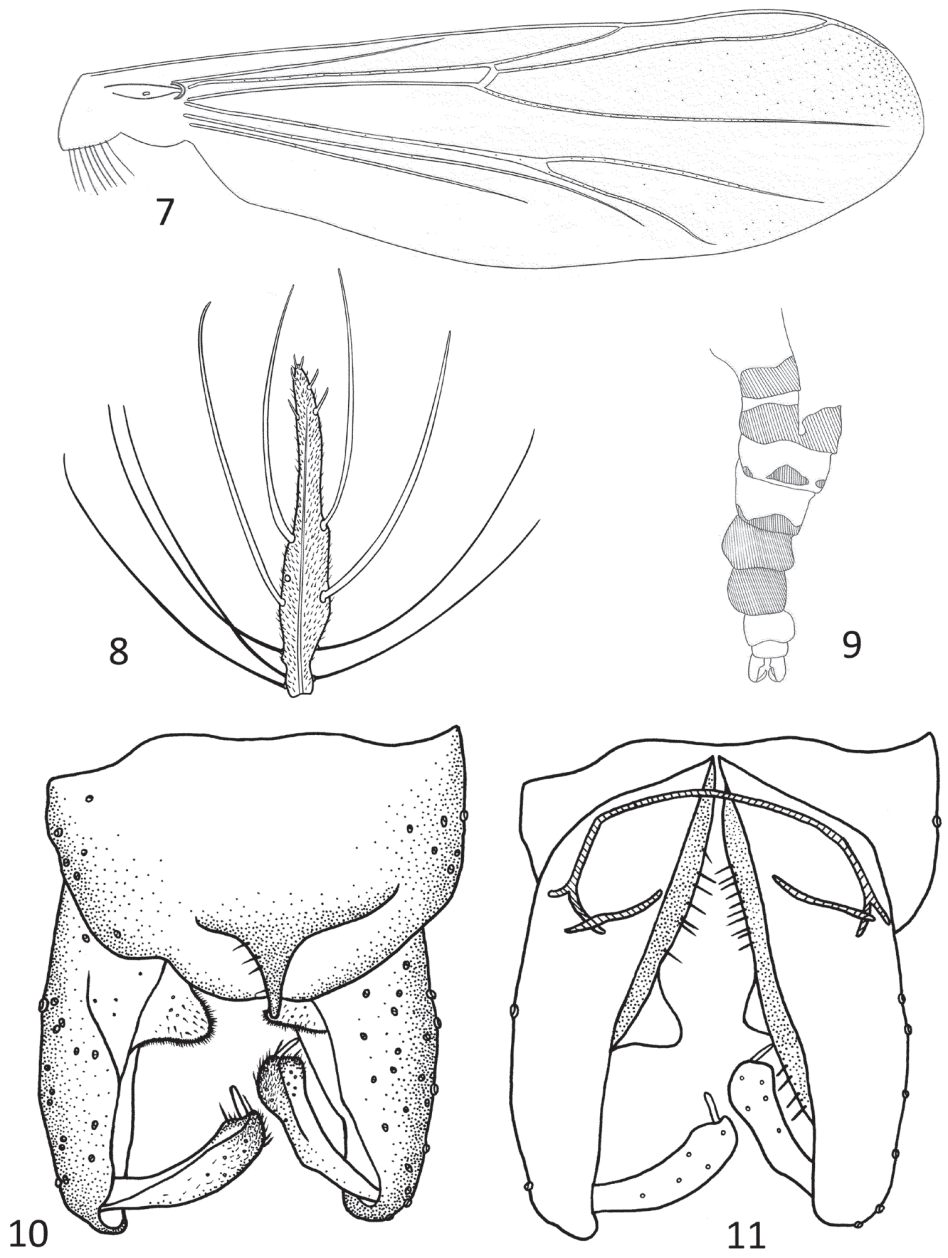
*Parametriocnemus vittaus* sp. n., male **7** abdomen **8** antenna**9** wing **10** hypopygium (dorsal view) **11** hypopygium(ventral view).

*Coloration*. Head, legs and antenna brown. Thorax light brown. Abdomen yellowish, tergites II–III with brown vita, 2/3 the width of the tergite, tergites IV–V with three brown patches on each tergite, and tergites VI –VII all brown (Fig. 7).

*Head*. AR 0.58. Ultimate flagellomere expand in the middle, tapering toward apex, with 4 long, curved sensilla chaetica subapically (Fig. 8). Temporal setae 9, including 4 inner verticals, 4 outer verticals and 1 postobital. Clypenus with 13 setae. Tentorium 110 µm long, 10 µm wide. Length of palpomeres (in µm): 23, 30, 85, 105, 160. Length ratio of palpomere 5/3 1.88.

*Wing* (Fig. 9). Anal lobe reduced. VR 1.22. Costal extension 120 µm long. Brachiolum with 1 seta, R with 20, R_1_ with 9, R_4+5_ with about 300, M without setae, M_3+4_ with 17 setae. Most of the wing membrane densely covered with setae; cell r_4+5_ with 141 setae, m_3+4_ with 13 setae. Squama with 8 seta.

*Thorax*. Antepronotum with 1 setae. Dorsocentrals 10, acrostichals 13, prealars not visible. Scutellum with 8 setae.

*Legs*. Spur of fore tibia 33 µm long, spurs of mid tibiae 23 µm and 20 µm long, of hind tibia 50 µm and 18 µm long. Width at apex of fore tibia 35 µm, of mid tibia 40 µm, of hind tibia 50 µm. Comb of 10 setae, shortest seta 28 µm long, longest seta 60 µm long. Lengths (in µm) and proportions of legs as in Table 5.

**Table 5.** Lengths (in µm) and proportions of legs segments of *Parametriocnemus vittatus* **sp. n.**, male (n=1).

	**fe**	**ti**	**ta_1_**	**ta_2_**	**ta_3_**	**ta_4_**
p_1_	620	670	540	264	194	132
p_2_	660	610	300	136	97	78
p_3_	690	750	410	180	154	90
	ta_5_	LR	BV	SV	BR	
p_1_	100	0.81	2.65	2.39	1.36	
p_2_	75	0.49	4.07	4.23	1.80	
p_3_	88	0.55	3.61	3.51	1.43	

*Hypopygium* (Figs 10–11). Tergite IX including anal point with 5 setae. Laterosternite IX with 6 setae. Anal point 38 µm long, 33 µm wide. Gonocoxite 208 µm long, inferior volsella triangular. Gonostylus 75 µm long, megaseta 13 µm long. HR 2.77, HV 3.4.

#### Remaks.

The new species is similar to *Parametriocnemus stylatus* in the structure of the hypopyium, while the body color is close to *Parametriocnemus scotti*. However, both *Parametriocnemus stylatus* and *Parametriocnemus scotti* lack brown vita on tergites II–VII.

#### Distribution.

The species was collected in Sichuan Province in Oriental China.

### Key to adult males of *Parametriocnemus* in China

**Table:** 

1	Ultimate flagellomere with 3–4 long, curved sensilla chaetica subapically	2
–	Ultimate flagellomere without long, curved sensilla chaetica subapically, sometimes with numerous short curved bristles	3
2	AR 0.31–0.46; ultimate flagellomere short, not expended in the middle; tergites II–VII without brown vita or patches; inferior volsella broadly rounded	*Parametriocnemus ornaticornis* (Kieffer)
–	AR 0.58; ultimate flagellomere long, expended in the middle, tapering towards apex; tergites II–VII with brown vita or patches; inferior volsella trangular	*Parametriocnemus vittatus* sp. n.
3	Inferior volsella broadly rounded; entire wing membrane densely clothed with setae	4
–	Inferior volsella triangular; basal half of wing membrane bare or at most with scattered setae in anal cell	5
4	Gonostylus with broad, transparent, preapical crista dorsalis	*Parametriocnemus lundbeckii* (Johannsen)
–	Gonostylus without transparent, preapical crista dorsalis	*Parametriocnemus stylatus* (Kieffer)
5	Anal point 80–143 µm long, extending well below posterior margin of tergite IX; squama with 8 setae; AR≤ 0.6 or ≥ 1.0; antenna without numerous curved bristles	6
–	Anal point short, 25–40 µm long, not extending below posterior margin of tergite IX; squama with 2–5 setae; AR 0.77–0.88; antenna with numerous curved bristles	*Parametriocnemus scotti* (Freeman)
6	AR 0.57; anal point 143 µm long, without setae; setae on terga not arranged in rows; acrostichichals in single, irregular rows; HV 4.3	*Parametriocnemus fortis* sp. n.
–	AR 1.03, anal point 80 µm long, with setae; setae on terga arranged in transverse rows; acrostichichals in two irregular rows; HV 3.12	*Parametriocnemus brundini* Sinharay et Chaudhuri

## Supplementary Material

XML Treatment for Parametriocnemus brundini

XML Treatment for Parametriocnemus fortis

XML Treatment for Parametriocnemus lundbeckii

XML Treatment for Parametriocnemus ornaticornis

XML Treatment for Parametriocnemus scotti

XML Treatment for Parametriocnemus stylatus

XML Treatment for Parametriocnemus vittatus
